# Supplementary material for: Effects of protein sources at sahur on anaerobic power and strength during Ramadan in combat sport athletes: A single blind, randomized, placebo-controlled, counterbalanced crossover study design
Source: PLoS One. 2026 Mar 13;21(3):e0340961. doi: 10.1371/journal.pone.0340961 (PMC12987419; doi:10.1371/journal.pone.0340961)
Supplement: Attachment — Submitted filename: Response Letter to reviewers.docx [file pone.0340961.s003.docx]

## ****Response to the Editor****

Dear Editor,

We would like to express our sincere gratitude for the opportunity to revise our manuscript and for overseeing the review process. We appreciate the constructive and insightful comments provided by all reviewers, which have significantly strengthened the clarity, methodological rigor, and overall quality of our work.

We are pleased to confirm that **all reviewer suggestions have been fully addressed**. The manuscript has been revised accordingly, including updates to the Study Design, Methods, Statistical Analysis, Results, and Discussion sections. Additionally, the **title has been shortened and improved in line with Reviewer #2’s recommendation**, resulting in a clearer and more concise presentation.

Should any further revisions or clarifications be required, we are fully prepared to respond promptly and thoroughly.

Thank you once again for your guidance and for considering our manuscript for publication.

Kind regards.

## ****Response to Reviewer #1****

We sincerely thank the reviewer for the careful evaluation of our manuscript and for the insightful comments, which substantially improved the clarity and methodological accuracy of the study. Below, we provide a point-by-point response, along with the corresponding revisions made to the manuscript.

## ****1. Crossover design and washout period****

***Reviewer comment:***
A 7-day washout period between each of the four conditions appears incompatible with completing the study “within the first two weeks of Ramadan.”

***Response:***
Thank you for identifying this important point. We agree that our original phrasing created an unintended inconsistency. The study did **not** use fixed 7-day washout intervals. In practice, we applied an **individualized washout period ranging from a minimum of 3 to a maximum of 7 days**, depending on each participant’s schedule. This allowed all Ramadan conditions to be completed within a 14-day window while still respecting the minimum metabolic recovery period required in crossover designs.

The manuscript has been revised to accurately describe this procedure:

“An individualized washout interval of **at least 3 and up to 7 days** was applied between conditions, enabling all Ramadan sessions to be completed within a 14-day period while preserving adequate metabolic washout.”

We thank the reviewer for helping us clarify this key methodological detail.

## ****2. Caloric imbalance between conditions****

***Reviewer* *comment:***
The placebo was described as 0.8 g/kg maltodextrin, which would not be isocaloric with the protein conditions.

***Response:***
We appreciate the reviewer drawing attention to this. The issue originated from a **writing error**, not from the experimental procedures. In the actual study, **all supplements—including the placebo—were administered at 0.4 g/kg**, making them **isocaloric by design**. The value “0.8 g/kg” appeared due to a misinterpretation during English translation of the Turkish draft, where “the same as the supplements” was mistakenly interpreted as “the sum of the two protein doses.”

We have corrected the manuscript accordingly, and we explicitly note the correction in the revision:

“For the placebo condition, participants ingested **0.4 g/kg maltodextrin**, matched isocalorically to the protein supplements.”

We thank the reviewer for helping us identify and correct this mistake.

## ****3. Timing of the control condition (fed vs. fasting duration)****

***Reviewer comment:***
Control testing was performed 3–5 hours after lunch, whereas fasting conditions were tested after 11–13 hours of fasting. Could this timing difference explain performance differences?

***Response:***
This is an important point and was revised extensively for clarity. The timing difference is **inherent to the study question**, not a methodological flaw. The purpose of the control session was to provide a **non-fasted reference**, reflecting athletes’ habitual training schedule outside Ramadan. It would not have been ethically appropriate to ask Muslim participants to break their fast during Ramadan to create a “fed control.”

Crucially:

- **All sessions—control and Ramadan—were conducted at exactly the same time of day (16:00–18:00)**.
- The only intentional difference was **fasting duration**, because the study aims to examine how **Ramadan-specific fasting load** interacts with nutrient timing.

We have revised the manuscript to clarify this rationale:

“The control session took place during the week before Ramadan, 3–5 hours post-lunch, providing a habitual non-fasted reference. This timing difference was intentional and reflects the central comparison of the study: performance in the fed state versus the prolonged fasting state characteristic of Ramadan.”

We also added a note in the Discussion acknowledging this as a **physiological, not methodological**, factor.

## ****4. ANOVA inconsistencies (Table 1)****

***Reviewer comment:***
The p-values for CMJ and Leg Press were inconsistent with their F-values.

***Response:***
We thank the reviewer for noticing this detail. This was indeed a **typographical error**: the value reported as **p = 0.979** should have been **p = 0.079**. The correct value matches the associated F statistic.

We have:

- corrected the value,
- re-checked all ANOVA outputs,
- ensured consistency throughout Table 1 and the Results section.

The raw dataset has also been submitted for transparency.

## ****5. Effect size reporting (Hedges’ g)****

***Reviewer comment:***
Some Hedges’ g values appeared implausibly large.

***Response:***
We fully agree with the reviewer’s concern. After re-evaluating our calculations, we determined that the previous values were inflated due to:

1. misapplication of pooled SD in a **within-subjects** design, and
2. an incorrect standardization step in the early version of the analysis.

We have now recalculated all effect sizes using the appropriate **Hedges’ g<sub>av</sub> (average-standardized)** method recommended for repeated-measures and crossover studies. The revised effect sizes now align exactly with the reviewer’s expectations. For example:

- Control vs. WPI (Wingate Peak Power)
  - Original (erroneous): g = 4.88
  - Corrected: **g*_aw_* = 1.29**

All effect sizes were recomputed and updated in the manuscript, and the Statistical Analysis section now clearly explains why **g<sub>av</sub>** was chosen over classical Hedges’ g or Cohen’s d.

We sincerely appreciate the reviewer’s careful attention, which greatly improved the statistical robustness of the study.

## *****General Response*****

We thank the reviewer for the constructive feedback. All methodological descriptions have now been clarified, statistical errors corrected, and effect size computations updated. We agree that these improvements substantially strengthen the manuscript and enhance its interpretability. We genuinely appreciate the reviewer’s expertise, which helped us refine the study into a methodologically robust contribution to the literature on nutritional strategies during Ramadan.

## ****Response to Reviewer #2****

We sincerely thank the reviewer for the constructive and thoughtful feedback. We appreciate the positive assessment of our study design, methodological rigor, and overall manuscript quality. We have carefully revised the manuscript in line with the reviewer’s recommendations. Below, we provide detailed responses to each point and highlight the corresponding changes made.

### **1. Title revision**

***Reviewer comment:*** The current title is lengthy; a more concise alternative is recommended.

***Response:***
We agree that the original title included excessive methodological detail. Following the reviewer’s suggestion, we adopted a shorter and clearer title:

**“Protein Intake at Sahur and Anaerobic Performance During Ramadan: A Randomized Crossover Study in Combat Sport Athletes.”**

This revised title maintains methodological transparency while improving readability.

### **2. Abstract – Streamlining statistical details**

***Reviewer comment:*** The abstract contains too many p-values and effect sizes; readability should be improved.

***Response:***
We revised the abstract to reduce statistical density. The abstract is now more concise and reader-friendly while still reflecting the study’s main findings.

### **3. Introduction – Highlighting the research gap**

***Reviewer comment:*** The novelty of directly comparing WPI and MC at sahur should be emphasized more clearly.

***Response:***
A new sentence was added to the final paragraph of the Introduction to strengthen the research gap and highlight the unique contribution of our study:

“Despite considerable interest in optimizing performance during Ramadan, no studies have directly compared fast-digesting WPI and slow-digesting MC consumed at sahur within a controlled crossover design, leaving an important gap regarding how protein type influences anaerobic performance following prolonged fasting.”

This addition clearly states the study’s novelty and scientific relevance.

### **4. Washout period justification**

***Reviewer comment:*** The 7-day washout period should be justified.

***Response:***
We revised the Methods section to clarify that the washout period was individualized between **3 and 7 days**, rather than a fixed 7 days, enabling all Ramadan sessions to be completed within 14 days without compromising washout adequacy.

### **5. Power analysis – Rationale for choosing f = 0.25**

***Reviewer comment:*** Provide justification for using a medium effect size in the a priori power analysis.

***Response:***
We added text explaining that selecting a medium effect size is a **conservative and commonly used approach** in repeated-measures crossover studies with limited prior data.

### **6. Equipment calibration clarification**

***Reviewer comment:*** Confirm whether calibration was repeated before each test day.

***Response:***
We updated the Procedures section to explicitly state:

“All equipment, including the ForceDecks system and dynamometers, was calibrated before each testing day to ensure measurement consistency.”

### **7. Rest interval conditions**

***Reviewer comment:*** Clarify whether athletes were monitored during the 10-minute rest period.

***Response:***
We added the following clarification:

“During the 10-minute rest intervals between strength tests, athletes remained seated under researcher supervision to standardize recovery and prevent unintended physical activity.”

### **8. Practical implications for coaches (Discussion)**

***Reviewer comment:*** Expand slightly on practical applications, especially regarding MC.

***Response:***
We incorporated a concise but meaningful paragraph into the Discussion and further emphasized this point in the Conclusions section:

“From a practical perspective, these findings suggest that athletes engaged in anaerobic-dominant sports may benefit from preferring micellar casein at sahur, as its slow digestion profile appears more effective in maintaining anaerobic power during prolonged fasting.”

This addition aligns directly with the reviewer’s recommendation and enhances applied relevance.

## *****Final remark*****

We are grateful for the reviewer’s insightful suggestions, which have substantially improved the clarity, methodological transparency, and applied relevance of the manuscript. All recommended revisions were addressed without altering the study’s results or interpretations.

## ****Response to Reviewer #3****

We sincerely thank the reviewer for the positive evaluation of our study and for acknowledging its novelty and relevance. We appreciate the constructive suggestions regarding additional variables such as age, gender, BMI, speed, flexibility, and biochemical markers (e.g., AST, ALT). While these factors are indeed valuable for future investigations, they were beyond the scope of the present study, which focused specifically on the acute effects of sahur-time protein intake on anaerobic power and strength in a controlled crossover design.

We agree that incorporating these physiological and biochemical variables would enrich future research and help broaden the understanding of performance responses during Ramadan fasting. We have added this point to the “Future Research Directions” section to reflect the reviewer’s helpful recommendations.

We thank the reviewer once again for the thoughtful comments.
